# Kaposi Sarcoma in an HIV-Negative Patient Following Temozolomide Chemoradiation Treatment for High-Grade Glioma

**DOI:** 10.7759/cureus.105840

**Published:** 2026-03-25

**Authors:** Zoobia Khan, Taylor C Boggess, Julia An, Walter Smithwick, Raafat Makary

**Affiliations:** 1 Pathology and Laboratory Medicine, University of Florida College of Medicine – Jacksonville, Jacksonville, USA; 2 Ophthalmology, University of Florida College of Medicine – Jacksonville, Jacksonville, USA

**Keywords:** eyelid tumors, hiv-negative kaposi sarcoma, human herpesvirus 8 (hhv8), post-chemotherapy complication, temozolomide

## Abstract

Kaposi sarcoma is a rare vascular neoplasm caused by human herpesvirus 8 (HHV8), commonly associated with human immunodeficiency virus (HIV) infection or iatrogenic immunosuppression. This report describes the case of a 44-year-old male who recently completed chemoradiation therapy for a high-grade midline glioma who presented with a right upper eyelid lesion. Wedge resection of the lesion was performed. Microscopic examination revealed a nodular proliferation of atypical medium-sized spindled cells with numerous vascular spaces and scattered mitotic figures. Immunohistochemical staining confirmed that the atypical cells were positive for HHV8, supporting the diagnosis of Kaposi sarcoma. This case may represent an exceptionally rare example of Kaposi sarcoma in a patient with immunosuppression resulting from chemotherapy for a previously diagnosed brain tumor. This case may help inform future investigations into the molecular mechanisms of HHV8-induced cellular transformations that lead to the development of Kaposi sarcoma and may also inform future conversations on the risks and benefits of different chemotherapy regimens in high-grade glioma patients.

## Introduction

Kaposi sarcoma is a rare vascular neoplasm of intermediate malignancy characterized by a proliferation of atypical spindled endothelial cells. It typically presents as a red, purple, or brown macule or patch on the epidermis or a mucous membrane that may or may not be painful. The patch may then progress to a raised plaque and eventually to a nodular tumor [1,2]. Cutaneous and localized lesions may be treated with surgical excision. However, if left untreated, the disease has the potential to progress to a systemic form, which carries a significantly worse prognosis and often requires systemic chemotherapy for treatment [3]. There are multiple subtypes of Kaposi sarcoma, all of which are associated with the infection of endothelial cells, monocytes, and B-lymphocytes by human herpesvirus 8 (HHV8) [2,4]. The different subtypes share similar immunohistochemical staining patterns, which allow them to be differentiated from other neoplastic skin lesions and other vascular neoplasms. The majority of subtypes are also associated with immunosuppression resulting either from disease, such as human immunodeficiency virus (HIV), or secondary to treatment for another condition.

Temozolomide is an alkylating agent widely used in the treatment of high-grade gliomas and other primary brain tumors. It has previously been shown to induce lymphopenia and functional immunosuppression, particularly when taken concurrently with corticosteroids [5]. Opportunistic infections and cancers attributable to infections have been reported in patients treated with temozolomide, but instances of Kaposi sarcoma in patients with temozolomide-induced immunosuppression are seemingly very rare [6-9]. Herein, we report a unique case of HHV8-positive Kaposi sarcoma of the eyelid in a patient who recently completed chemoradiation for a high-grade midline glioma.

## Case presentation

**Figure 1:**
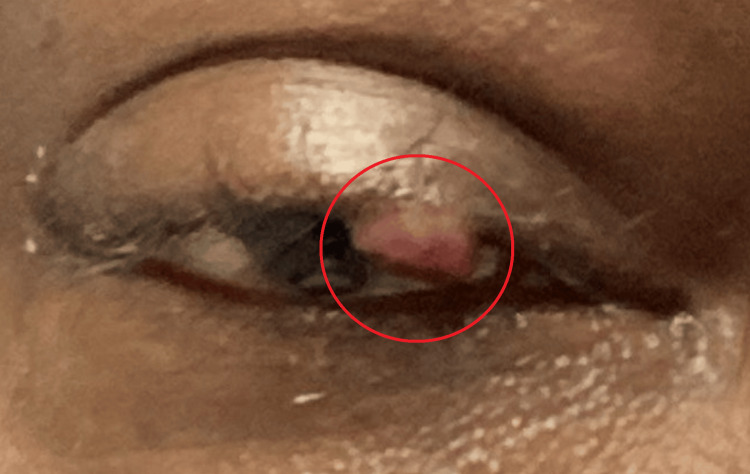
Eyelid lesion at presentation A 7 mm round, ulcerated, painless lesion without drainage on the right upper eyelid has been present for two months (red circle).

A 44-year-old African-American male with a past medical history of type 2 diabetes mellitus, prior cocaine use, and a 28-pack-year tobacco smoking history presented to ophthalmology for a right upper eyelid lesion. The patient had recently completed a chemoradiation regimen for high-grade midline glioma consisting of Proton radiation therapy for six weeks, followed by six consecutive 28-day cycles of temozolomide monotherapy. At the time of clinical presentation, the patient was also taking prescribed oral Decadron 4 mg once daily. The eyelid lesion had been present for at least two months at the time of presentation. The lesion consisted of a 7 × 7 mm, roughly circular, skin-colored lesion with central ulceration that had been present for approximately two months (Figure 1). The lesion was painless and without drainage. It showed no improvement after treatment with topical erythromycin. Wedge resection of the lesion was performed, and the tissue was submitted for pathology. A complete metabolic panel (CMP) and complete blood cell count (CBC) from less than one week following the resection surgery showed mild anemia, elevated blood glucose, and mildly elevated liver enzymes, but showed no leukopenia or other significant indications of immunosuppression (Table 1).

**Table 1:** Laboratory results Select results from the patient's complete metabolic panel (CMP) and complete blood cell count (CBC) collected less than one week following resection of the lesion

Lab value	Result	Reference range
Hemoglobin (g/dL)	13.9	14.0-18.0
Mean corpuscular volume (fL)	92.8	82.0-101.0
White blood cell count (1000/mm^3^)	5.91	4.5-11
Absolute lymphocyte count (1000/mm^3^)	2.02	0.90-3.00
Absolute neutrophil count (1000/μL)	3.27	1.4-7.5
Platelet count (1000/μL)	212	140-440
Blood glucose (mg/dL)	159	71-99

**Figure 2:**
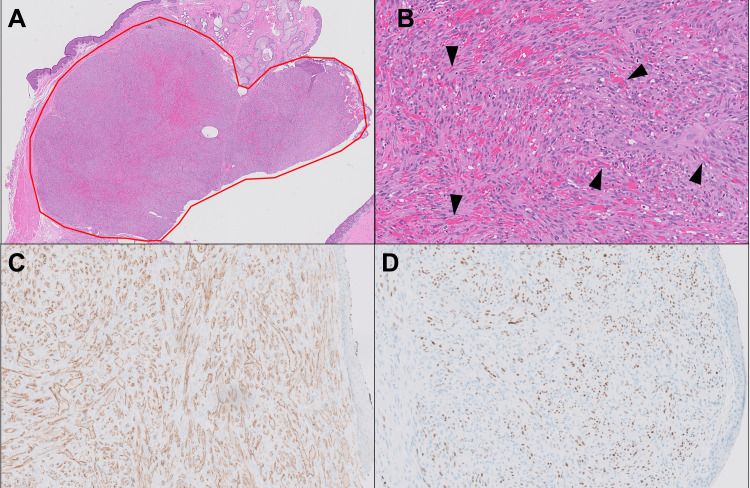
Microscopic morphology and immunohistochemistry Hematoxylin and eosin (H&E)-stained section showing a nodular proliferation of atypical spindle cells forming slit-like vascular channels with scattered mitotic figures at 5X (A, red circle) and 63X (B) magnification (black arrows showing examples). Immunohistochemical stains showing CD31 positive (C) and HHV8 (LANA-1) positive (D).

Initial differential diagnoses based on the gross morphology and clinical history included hemangioma, chalazion, hordeolum, basal cell carcinoma, sebaceous skin tumor, and Kaposi sarcoma. Hematoxylin and eosin (H&E)-stained slides of the serially sectioned lesion showed a nodular proliferation of atypical medium-sized spindled cells with irregular nuclear contours and occasional prominent nucleoli in association with numerous vascular spaces and scattered mitotic figures (Figure 2A, 2B). Immunohistochemical staining for CD31 highlighted atypical vascular channels within the tumor (Figure 2C). The atypical appearing cells showed strong positive nuclear staining for HHV8 (LANA-1; Figure 2D). Additional immunohistochemical staining further characterized the vascular nature of the tumor (results in Table 2).

**Table 2:** Immunohistochemistry results

IHC	Result
HHV8 (LANA-1)	Positive in a subset of atypical cells
CD34	Highlights numerous vessels
CD31	Highlights numerous vessels, similar to CD34
SMA	Highlights numerous vessels
S100	Rare positive cells
Desmin	Negative in atypical cells

Based on the histologic features and immunohistochemical results, and in light of the patient’s clinical history and HIV-negative status, the final diagnosis of iatrogenic Kaposi sarcoma was made. Following the diagnosis, the patient has continued to follow up with ophthalmology, and no recurrence or complications from the eyelid resection have been reported at more than six months post-surgery. The patient has also continued to follow up with oncology for ongoing monitoring and treatment for high-grade glioma.

## Discussion

This case represents a rare example of iatrogenic Kaposi sarcoma arising in a patient with no HIV history following chemoradiation therapy. There are currently at least four subtypes of Kaposi sarcoma (Table 3), but all subtypes are caused by HHV8 infection, typically transmitted through direct contact with saliva, blood, or other body fluids. This case most likely represents an example of the iatrogenic subtype of Kaposi sarcoma, which is seen most commonly in immunosuppressed solid-organ donation recipients who contract HHV8, but can also be seen in patients with immunosuppression from chemotherapy and/or radiation or other immune/autoimmune disorders [10-14]. 

**Table 3:** Kaposi sarcoma subtypes

Subtype	Features	Patients
Classic	Most commonly presents with lower extremity lesions [11], seen in the setting of immunosenescence with a decreased lymphocyte count.	Typically elderly men, initially associated with Mediterranean and Eastern European Jewish descent.
Endemic	Occurs predominantly in sub-Saharan Africa, associated with malnutrition-associated immunosuppression [12]	HIV-negative, men > women
Epidemic	AIDS-related immunosuppression, most common type in the United States, typically the most aggressive subtype, usually presenting with a more disseminated stage of disease [10,13]	HIV-positive, highest risk group is African-American and Hispanic men who have sex with men.
Iatrogenic	Immunosuppression due to solid organ transplants, treatment for other malignancies, or immune/autoimmune disorders [14].	Organ transplant recipients most common.

According to the patient, the eyelid lesion first became noticeable between his fourth and fifth cycles of temozolomide chemotherapy, and the lesion was removed less than one month after his sixth and final cycle. Although Kaposi sarcoma lesions of the eyelid have previously been reported [15], the case of Kaposi sarcoma presented in this report is unique in its presentation, given that it is a case of the iatrogenic subtype. Temozolomide, as a chemotherapeutic agent, has been implicated in lymphopenia and myelosuppression [5]. However, a CBC from around the time at which the lesion appeared showed no significant white blood cell abnormalities (Table 1). Although cases of Kaposi sarcoma have been reported in immunocompetent patients [6,7,16], the timing of the appearance of the Kaposi sarcoma in this case, coinciding with the patient’s temozolomide therapy, does suggest that the therapy was associated with a level of immunosuppression that allowed for the HHV8 infection to manifest. Even if the number of lymphocytes in circulation did not noticeably decrease, their normal functioning and ability to respond to a viral infection may have been diminished in this patient. In addition, the patient’s clinical history of type 2 diabetes mellitus and long-term treatment with corticosteroids may have also contributed to the immunosuppression required for the transmission of HHV8 and development into Kaposi sarcoma.

This report illustrates the methods by which a pathologic diagnosis of Kaposi sarcoma can be reached. The presentation of Kaposi sarcoma can vary significantly between different patients, and multiple techniques are often necessary to reach a diagnosis. In addition to positive immunostaining for HHV8-associated protein LANA1 in atypical cells [17,18] and the positive staining of CD34, CD31, and SMA within vascular channels, the morphology of fascicles of spindled cells with extravasated red blood cells and scattered chronic inflammatory cells allows for differentiation from other skin malignancies [2,3]. The presence of atypical nuclear features and increased mitoses aided in the elimination of non-malignant entities, such as spindle-cell or tufted hemangioma, from the differential. Entities such as basal cell carcinoma and sebaceous skin tumors, both of which can present as nodular eyelid lesions, typically feature more basaloid appearing malignant cells [19]. Although it was not implemented in the diagnosis of this patient, polymerase chain reaction (PCR) can also be used to amplify and detect HHV8-specific viral DNA in cases of negative LANA1 immunostaining [20]. Additional diagnostic methods are likely to be developed as HHV8 and its manifestations continue to be investigated.

## Conclusions

This report describes a case of Kaposi sarcoma arising in a patient who recently received treatment for a high-grade glioma. Although rare, Kaposi sarcoma can arise in patients undergoing chemoradiation therapy. The case presented in this report is unique in its presentation with regard to the patient’s clinical history, combined with the location of the tumor on the patient’s eyelid. This case underscores the need for vigilance when evaluating atypical skin lesions in patients undergoing temozolomide therapy and highlights the diagnostic value of HHV8 immunohistochemistry in spindle-cell vascular proliferations. Future investigations into the infectious processes of HHV8 and how these processes are impacted by temozolomide and other chemotherapeutic agents may inform future cancer treatment plans and lead to earlier detection of Kaposi sarcomas when they occur in cancer patients.
